# Transcranial Direct Current Stimulation (tDCS) in the Treatment of Youth Depression: Integrating Literature Review Insights in a Pilot Clinical Trial

**DOI:** 10.3390/jcm14093152

**Published:** 2025-05-01

**Authors:** Heidi Ka Ying Lo, Suet Ying Yuen, Iris Wai Tung Tsui, Wing Fai Yeung, Jia Yin Ruan, Corine Sau Man Wong, Joyce Xu Hao Jin, Chit Tat Lee, Ka Fai Chung

**Affiliations:** 1 Department of Psychiatry, The University of Hong Kong, Hong Kong, China; u3012372@connect.hku.hk (S.Y.Y.); u3585363@connect.hku.hk (J.X.H.J.);; 2 School of Nursing, the Hong Kong Polytechnic University, Hong Kong, China; 3 Rory Meyers College of Nursing, New York University, New York, NY 10010, USA; jr7121@nyu.edu; 4 School of Public Health, The University of Hong Kong, Hong Kong, China; 5 Department of Psychiatry, Queen Mary Hospital, Hong Kong, China

**Keywords:** depression, youth mental health, transcranial direct current stimulation, systematic review, clinical trial, treatment, youth depression, tDCS feasibility study

## Abstract

**Highlights:**

**What are the main findings?**

- This study identified transcranial direct stimulation (tDCS) as a promising and feasible treatment modality for youth depression. A systematic review conducted up until 20 November 2024 identified fourteen eligible registered/ published studies in tDCS for youth depression. Among the limited clinical data available, two trials demonstrated substantial symptom improvement. However, recruitment challenges and high risks of bias underscore the need for robust evidence supporting the feasibility of conducting tDCS RCTs in this population.
- This pilot trial demonstrated high session attendance and retention rates with no dropouts or serious adverse events during the five-day, 30 min, 2 mA tDCS protocol.
- Decentralised administration of tDCS required prompting in some cases and may have introduced variability in adherence.

**What are the implications of the main findings?**

- tDCS has the potential as a safe and acceptable intervention for youth depression
- First study to review and pilot youth tDCS implementation
- Provides practical insights into translating neuromodulation reviews into a pilot trial
- Although not powered to detect efficacy, it offers a framework for designing future trials
- Emphasizes need to evaluate long-term safety- specifically the absence of unintended outcomes of tDCS.

**Abstract:**

**Background**: Youth (ages 16–25) is a key window for mental health interventions, as depression rates significantly increase during this developmental stage. However, transcranial direct current stimulation (tDCS) application in youth depression remains underexplored. To reduce the uncertainty of a future trial, we conducted a review and a pilot randomised controlled trial (RCT) of tDCS for youth depression. **Methods**: Following the PRISMA guidelines, the first part of this study was a review across databases including PubMed, MEDLINE, PsychInfo, CINAHL, Open Access Theses and Dissertations (OATD), WanFang Data, Chinese Medical Journal, and clinical trial registries up to 20 November 2024, on tDCS treatment for youth depression. The second part of this study was a double-blind pilot RCT assessing feasibility, by comparing active tDCS (five daily 30 min 2 mA anodal tDCS applications over the left dorsolateral–pre-frontal-cortex (DLPFC) with sham tDCS. Feasibility outcomes included recruitment, data collection, attendance, retention and randomisation. Outcomes also included depression severity using the Hamilton Depression Rating Scale (HDRS), safety, tolerability, acceptability, and adequacy of blinding. Mann–Whitney U tests were used for between-group comparison. **Results**: Fourteen eligible studies were identified, with a pooled HDRS reduction of −9.6 (95% CI: −11.2 to −8.1, *p* < 0.001), though high risks of bias indicated a research gap. Using parameters derived from the review, we conducted a pilot RCT in which 20 youths were screened and 8 were randomised (aged 16–24; 3 females, 5 males). All randomised participants completed their assigned sessions without dropout or protocol discontinuations. Blinding was adequate, and participants’ willingness to engage improved over time. Both groups showed reductions in HDRS, with a greater mean reduction in the active group (−4.75 ± 2.96) compared to the sham group (−3.75 ± 3.78). No serious adverse events occurred, with only mild headaches and tingling reported. The tolerability profile was comparable. However, the decentralised administration of sessions may have introduced inconsistent tDCS applications. **Conclusions**: This review highlights a lack of RCTs on tDCS for youth depression. Our pilot trial demonstrates the feasibility of a sham-controlled design in youth depression, justifying larger-scale trials to evaluate the efficacy of tDCS in this population.

## 1. Introduction

### 1.1. Background

Depression is a common, chronic, and impairing disorder with first onset often occurring during adolescence [1] and affecting close to a quarter of all adults during their lifetime [2]. Worldwide, adolescence and early adulthood represent critical developmental stages characterised by significant emotional, cognitive, and social changes that can increase vulnerability to depression and suicidality [3]. Youth depression collectively contributes to a staggering 21.5 million disability-adjusted life-years [4], representing a considerable healthcare burden and a pressing problem to society. While guidelines and randomised controlled trials (RCTs) support the use of medication and psychotherapy as a standalone or combination treatment for major depressive disorder (MDD) in youths, a large proportion do not respond to either medication or psychotherapy [5]. Up to 60% do not respond to evidence-based psychotherapies, and the efficacy of antidepressants remains controversial [6]. Antidepressant medication is also associated with an elevated risk of suicidality among youths [7]. Psychotropic adherence in this population is typically poor [8] and limited by side effects [9], including hidden bipolarity [10], making medication-based treatment unappealing for this group [11]. There is therefore an unmet need for new, effective treatment options for depression that are safe and can be tolerated by youths [12]. Studies have revealed differences in the neuronal structure and chemical pathways between depressed youths and non-clinical cohorts, that may be resolved with neuromodulatory interventions [13].

A promising neuromodulatory intervention is transcranial direct current stimulation (tDCS), with its acute effect being a physiological change that reduces the threshold of membrane polarisation and increases synaptic excitability. tDCS is a non-invasive neurostimulation technique in which a weak, direct electric current (amplitude < 2 mA) is applied through electrodes placed on the scalp. tDCS induces physiological effects by changing the threshold of membrane polarisation, thereby affecting synaptic excitability. Unlike other modalities of neuromodulation like transcranial magnetic stimulation and electroconvulsive therapy, tDCS is portable, inexpensive, and easily administered, which enhances its scalability and allows for safe self-administration [14,15]. In general, anodal cortical stimulation enhances excitability, whereas cathodal stimulation reduces it [16]. The result of this tDCS paradigm is plasticity-related changes that modulate NMDA-glutamatergic receptors, promote synaptic LTP, and facilitate adaptative neuronal organisation [17]. Recent work has suggested that frontolimbic development in depressed youths is delayed or aberrant [18]. Therefore, the judicious application of non-invasive brain stimulation techniques may present a promising opportunity for durable interventions in youths with MDD [12].

While tDCS was shown to be a promising augmentative treatment for MDD with meta-analytic evidence [19], its safety profile in youths has also been favourably evaluated [20], typically associated with only mild adverse effects such as itching, tingling, mild burning, or pain sensations [21]. Despite these positive attributes, the feasibility and RCT design of tDCS trials in youth depression remain largely unexplored. A meta-analysis covering up to 1-July-2022 reported no trials or case reports have investigated tDCS in youths with depression, and hence no conclusion could be drawn on the effectiveness of tDCS on youth depression [22]. However, this systematic review included only three databases and restricted the language of articles only to English or German, potentially overlooking ongoing or unpublished trials that might offer valuable insights into the efficacy and utility of tDCS in treating youth depression [22]. Although their review conducted an additional search of two trial registries to identify unpublished studies, no registered trials had been identified. Utilising the search engine to explore within the trial registries on 26 December 2023, at least four registered trials in tDCS in youths were retrieved with completed plural “registrations dating back to 2022. Since clinical studies have been scarce in this field, these unpublished trials will provide valuable information to other researchers for designing RCTs of tDCS for youth depression.

To address this research gap, the first part of this study was a systematic review that included a further comprehensive search of published and non-published studies (initial search on 26 December 2023, updated search on 20 November 2024). The second part of this study was a pilot trial which aimed to integrate the insights into the design of an RCT investigating the effects and feasibility of tDCS treatment in youth depression.

### 1.2. Review

#### 1.2.1. Search Strategy

The protocol for this review was registered on the PROSPERO international prospective register of systematic reviews (CRD42024621837). During our initial search, we found that four of the eligible registered trials were recorded in the Chinese Clinical Trial Registry (ChiCTR). To determine if there were any published studies related to these trials, we included WanFang Data and the Chinese Medical Journal as databases in our search strategy. They were the nationwide database containing academic research of Chinese texts. Following PRISMA guidelines (refer to Supplementary Table S1) [23], eligible studies were identified by search databases, including PubMed, MEDLINE, PsychInfo, CINAHL, Open Access Theses and Dissertations (OATD), WanFang Data and the Chinese Medical Journal from inception to 20 November 2024. To identify the ongoing/unpublished trials and access the methodological design of the protocol, we searched the World Health Organisation International Clinical Trials Registry Platform (ICTRP) registry, the National Institute of Health (NIH) registry, the European Union Clinical Trials Register, and the International Standard Randomised Controlled Trials Number (ISRCTN) registry. We used the following search terms: (transcranial direct current stimulation or tDCS) AND (young people, child, adolescent, young adult, youth, boy, girl, and paediatric) AND (depression, depressive, MDD, major depressive episode, mood disorder). Relevant review articles and reference lists of the included studies were also searched. No language restrictions were set. Articles were screened by two independent assessors (KY and SY) until a consensus on the inclusion or exclusion of an article was reached. A third reviewer (KF) made a judgement when disagreement was not resolved by discussion. Data extraction includes publication year, study type, sample size, participant characteristics, details of the intervention and control conditions, primary outcome measures (changes in depressive symptoms), and adverse effects. The risk of bias assessment in this study was conducted using the Cochrane Risk of Bias 2 (RoB 2) tool for clinical trials. Discrepancies were resolved by a consultant with a third reviewer (KF).

#### 1.2.2. Eligibility Criteria

We included all randomised and non-randomised trials, case reports and registered trials of tDCS in youths under 25 years of age at enrolment with depression or depressive symptoms. Given the relatively novel application of tDCS in youth populations, many studies are still in the early stages, such as registered trials or exploratory case reports, and have not yet undergone peer review or been published. This inclusive approach was adopted to address the limited availability of high-quality studies in this emerging area of research while promoting transparency, rigour, and the identification of future research priorities in the application of tDCS for youth depression. The intervention being assessed is tDCS. Other types of neuromodulation, such as transcranial magnetic stimulation (TMS) or electroconvulsive therapy (ECT), were not included in this review. The comparators included the following: sham tDCS, defined as receiving less than 1 min of tDCS current intended to simulate the sensation of active tDCS without delivering any therapeutic stimulation, and standard treatment, defined as antidepressant medications or psychotherapy. We included studies regardless of their selected outcome measurements. The primary outcomes were feasibility in terms of safety (e.g., presence of serious adverse effects, premature termination of treatment) and efficacy (e.g., changes in depression severity, as measured by the score on the Hamilton Depression Rating Scale, HDRS, from baseline to the endpoint of treatment). Articles not restrictive of language were included. The number of initial hits was recorded. After removing duplicates, the abstracts of all identified articles were screened based on pre-defined inclusion criteria. If records were excluded, the reasons for exclusion were recorded. In case the information provided in the abstract was insufficient, the full text was screened. The eligible population was limited to youths who were clinically diagnosed with depression or have depressive symptoms as assessed by questionnaires. Studies were excluded if they were secondary research.

#### 1.2.3. Review Results

**Figure 1:**
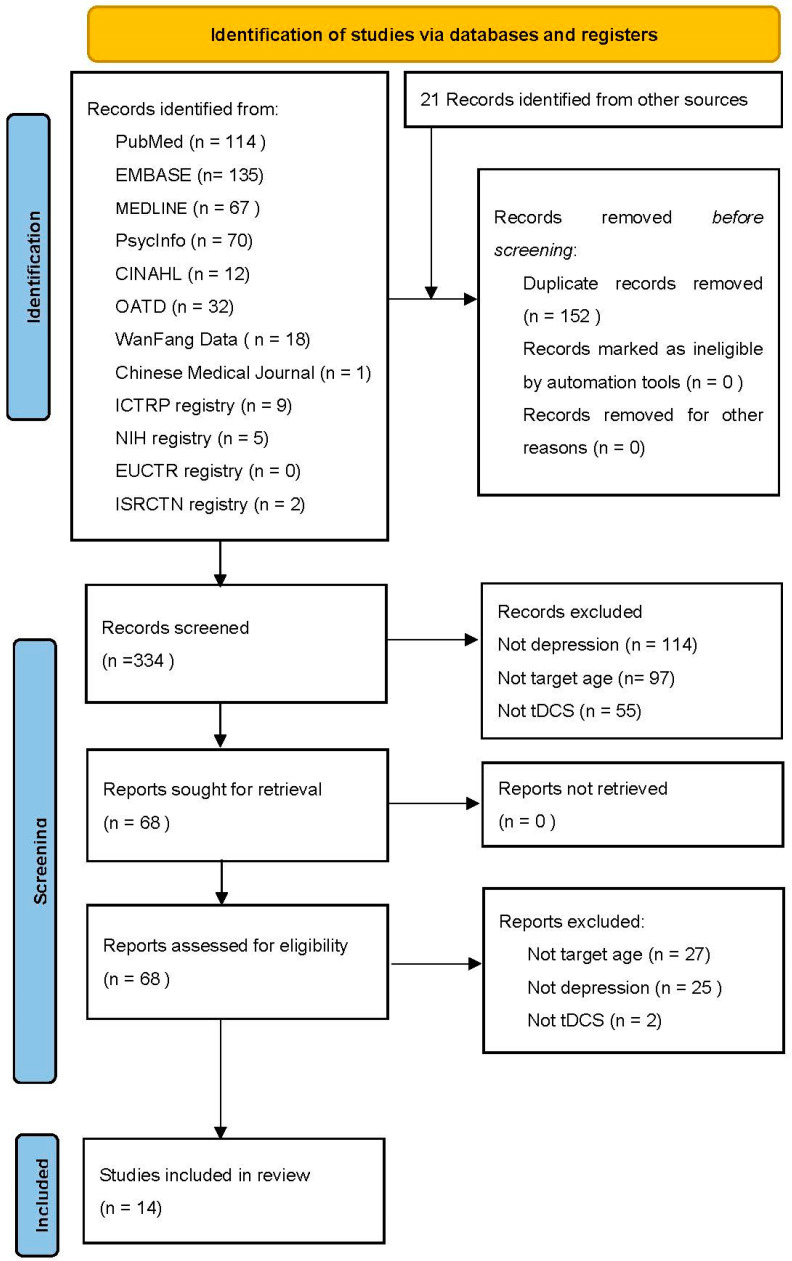
PRISMA flow diagram.

The initial search generated 486 studies from databases and registries, as seen in PRISMA (Figure 1). After removing duplicates, 334 papers remained for title and abstract screening. In total, 68 records were assessed for eligibility (52 original research papers, 16 trial registrations), and 14 remained for review. The majority were excluded due to individuals not having depression and not being targeted at the appropriate age. Of the identified studies, two studies were published clinical trials; four studies were case reports; and eight studies were clinical trials registered in registries.

Details of the included studies are summarised in Table 1. The included studies on tDCS protocols vary in stimulation paradigm, session count (ranging from 2 to 20), intensity (1.2 mA to 2 mA), and duration (20 to 30 min). Case reports and ongoing trials primarily utilize tDCS with anodal stimulation at the left DLPFC and cathodal simulation at the right DLPFC, including personalised high-definition tDCS (HD-tDCS) 4 × 1 ring montage setups. Control interventions in ongoing trials include sham tDCS alone, sham combined with mindful breathing, fluoxetine, and interpersonal or sandplay therapy. Most of the studies were published within the past five years, suggesting the rapid growth of tDCS research in this field.

#### 1.2.4. Clinical Trials

Two clinical trials which were both conducted in China showed promising results, though the studies presented certain limitations in their design and reporting. One sham-controlled trial (*n* = 66) was conducted in China and its report was published in 2017 [24]. The trial recruited youths aged 10–17 with a diagnosis of MDD. The intervention group offered 15 sessions of 20 min 1.2 mA tDCS augmented with sandplay therapy. Notably, the anodal stimulation was applied at the primary somatosensory cortex, which differs from the conventional placement at the left DLPFC. The results indicated significant improvements in depression scores, as measured by the Hamilton Depression Rating Scale (HDRS), with statistical differences noted at week 2, 4, and 8 (*p* < 0.001). However, the documentation of the study lacked details on randomisation and allocation methods, the setup of the sham condition, the evaluation of adverse effects, and dropout rates, which challenges its classification as an RCT. Another open-label study (*n* = 28) included youths aged 14–17 with major depressive episodes, including those with major depression or bipolar affective disorder [25]. This research investigated the effects of HD tDCS with anodal stimulation at the left DLPFC, delivering 20 sessions of 20 min tDCS at 2 mA. The pooled analysis demonstrates a significant reduction in HDRS scores following tDCS treatment in youths with depression, with an overall score of −9.6 (95% CI: −11.2–−8.1, *p* < 0.001). The risk of bias assessment for the included clinical trials revealed some concerns (refer to Supplementary Table S2). The sham-controlled trial [24] lacked detailed information on randomisation and allocation methods, raising potential bias in the randomisation process (D1), while the open label study [25] presented issues related to missing outcome data (D3) and the selection of reported results (D5). Both studies showed limitations in reporting adverse events and dropout rates, which impacted the overall risk of bias evaluation.

#### 1.2.5. Case Reports

We evaluated four case reports from peer-reviewed journals and conference abstracts involving individuals aged between 16 and 23 years from India and the United States. Two case studies from journals reported on two youths aged at 21 and 23 years old from India, respectively, who both completed 10 sessions of 2 mA on the left DLPFC [24,25]. One of them, a 21-year-old medical student with bipolar depression, had a 30% reduction in the HDRS score after 10 sessions of tDCS. It was reported that there was no rebound of manic symptoms as assessed by the Young Mania Rating Scale [26]. The other, a 23-year-old youth experiencing pregnancy, reported a reduction in the HDRS score from 18 to 5, at the post-1-month follow-up assessment after 10 sessions of tDCS [29]. Both individuals reported no adverse effects, suggesting safe application of the technique. Two case studies from conference abstracts included two youths from India and the United States [27,28]. In the case study from India, 20 sessions of tDCS were delivered to a youth aged 16. A significant reduction in the HDRS-17 score from 15 to 8 was reported [28]. It was interesting to note that the trial of tDCS was carried out after the unsuccessful treatment of ketamine infusion in a treatment-resistant depression case. In the case study from the United States, two sessions of 20 min 1.5 mA tDCS were self-administered by a youth aged 19 with a concurrent diagnosis of major depression and multiple sclerosis [27]. The Beck Depression Inventory revealed a significant clinical improvement, and the self-administered tDCS was shown to be safe and tolerable.

#### 1.2.6. Ongoing Unpublished Clinical Trials

We identified eight registered clinical trials focusing on tDCS for depression in youths. Of the eight registered trials, five ongoing RCT studies (ChiCTR2400079464; DRKS00027066; clinicaltrials.gov; NCT04780152; clinicaltrials.gov; NCT05498441; clinicaltrials.gov; NCT0606165), one completed RCT study (clinicaltrials.gov, NCT03897699), one ongoing observational study (ChiCTR200003950), and one withdrawn open-label study (clinicaltrials.gov; NCT03368469) were identified. The withdrawn study (clinicaltrials.gov; NCT03368469), targeting children and young people aged 10–21 with epilepsy and depressive disorder, faced recruitment challenges, resulting in its closure without enrollment completion. This study investigated the effect of 10 sessions of 20 min 1 mA tDCS over left DLPFC, with the measure of depression severity serving as a primary outcome measure. The only completed study (clinicaltrials.gov; NCT03897699) also encountered recruitment issues, completing with only 36 of the anticipated 68 participants. This study combined a 5-week mindful breathing training with a 2-week initial tDCS phase (2 mA, 20 min sessions targeting the left DLPFC) in the United States, aiming to assess the additive effects of tDCS on mindful breathing. The participants were young females with a mean age of 20.2 years living in the United States. The study reported its completion in 2023; it aimed to provide evidence on the safety and tolerability of tDCS in young females, but the hypothesis question was on the additive effect of tDCS on mindful breathing training instead of tDCS treatment. Subsequently, the severity of depressive symptoms was assessed by the Montgomery Asberg Depression Rating Scale at baseline and upon completion of mindful breathing training at week 5. The five ongoing RCTs investigated the effectiveness of tDCS in youth depression; all of them were ongoing by the time this report was prepared. One case–control observation study (ChiCTR200003950) was recruiting youths aged 10–23, with 11 primary outcome measures, 1 of them being HDRS. The outcome measure related to safety and tolerability was the McGill Pain Scale (MPF-SF). Otherwise, about half of these trials did not report on evaluating safety, tolerability, or acceptability, highlighting a gap in comprehensive evaluation. Aligning with existing theories, the methodological design of the tDCS protocol in youths with depression was 20 to 30 min 2 mA anodal stimulation over the left DLPFC. Recruitment difficulties have been a notable challenge across multiple studies.

### 1.3. Integrating Review Insights into a Pilot Trial

The studies identified in this review underline the potential of tDCS as a feasible treatment modality for youth depression, demonstrating substantial symptom reductions in the two completed clinical trials. However, recruitment challenges and high risks of bias underscore the need for robust evidence supporting the feasibility of conducting tDCS RCTs in this population. While the integration of fMRI with tDCS represents an important methodological advancement that allows for precise correlation between stimulation parameters and neural activity [30], establishing basic feasibility parameters must precede more resource-intensive combined approaches, especially in vulnerable youth populations where recruitment and retention challenges are already significant.

To this end, in our study, we conducted a pilot double-blind (participant, outcome accessor) sham-controlled trial. Given the demonstrated efficacy of short-term tDCS applications in prior studies, our RCT adopted a five-day, 30 min, 2 mA tDCS protocol to ensure compliance and practicality in a youth population. In adults, 2 mA stimulation produces optimal cortical excitability enhancements with after-effects persisting up to 24 h post-stimulation [31]. For youths, a comprehensive safety review documented 864 active tDCS sessions (0.5–2 mA) for children and adolescents aged 6–17 years, confirming tolerability and acceptability for protocols using up to 2 mA for 20 min across 1–20 sessions [32]. This approach aligns with the emerging consensus on non-pharmacological interventions for youth depression, particularly in youth where pharmacological options may be limited or poorly tolerated, underscoring the need for standardised, evidence-based protocols with youth-specific evidence [33]. The primary focus was on assessing feasibility outcomes, including recruitment, retention, adherence, blinding integrity, and the practicality of measuring clinical and patient-reported outcomes. Secondarily, we aimed to investigate the tolerability of a domain where previous research has shown a systematic gap in evaluation. We addressed this by implementing an Adverse Events Questionnaire developed from systematic reviews of tDCS trials [21], alongside measures of adherence, reasons for dropout, and participant-reported acceptability and willingness to engage in treatment.

Considering the existing literature and preliminary evidence, we hypothesised that tDCS would be well tolerated with no significant difference in tolerance between active and sham stimulations. Furthermore, the trial would achieve high retention rates, adequate adherence—specifically, participants completing at least 80% of scheduled tDCS sessions with correct electrode placement and stimulation parameters [34]—and successful implementation of blinding procedures, supporting the feasibility of conducting larger-scale RCTs.

## 2. Materials and Methods

### 2.1. Study Design

**Figure 2:**
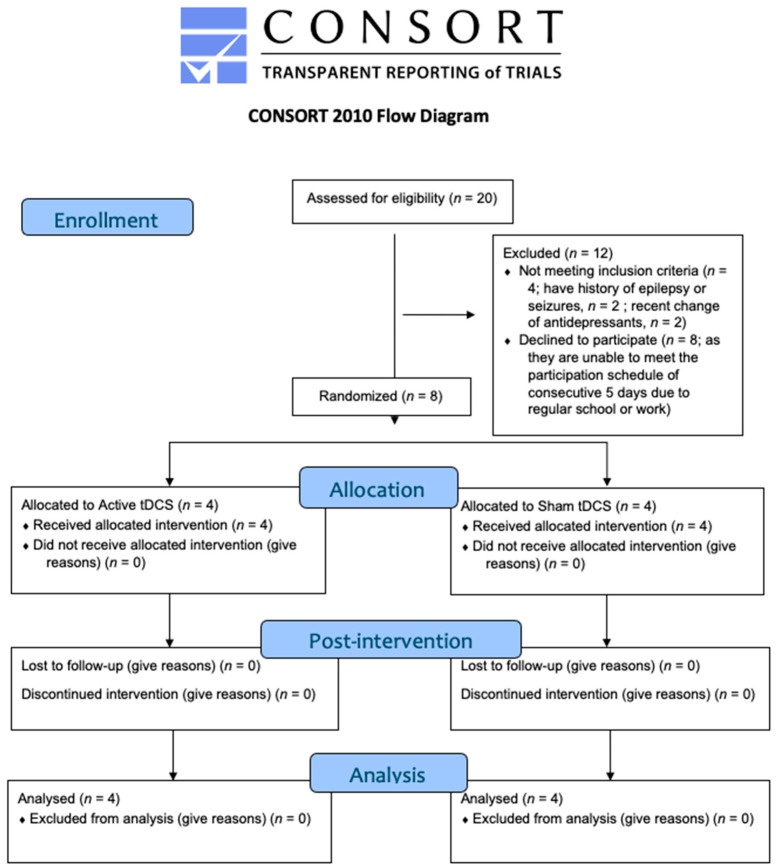
CONSORT flow diagram of the study trial.

The trial was prospectively registered in clinicaltrials.gov (ID: NCT06518642). The study was approved by the Institutional Review Board of the University of Hong Kong/Hospital Authority Hong Kong West Cluster (HKU/HA HKW IRB Reference Number: UW 24–035). The Consolidated Standards of Reporting Trials (CONSORT) flowchart is presented in Figure 2, and the CONSORT checklist for pilot or feasibility randomised trials is presented in Supplementary Materials Table S3. This is a randomised, double-blind (participant, outcome accessor), sham-controlled trial with two assessment time points: baseline (T0) and post-intervention (T1). Participants were randomly assigned to receive either active or sham tDCS by the principal investigator, with sessions conducted over five consecutive days. The randomisation ratio was 1:1 between the two groups.

### 2.2. Participants

Participants were recruited from July to October 2024 and the study was completed in November 2024. Youths diagnosed with MDD were recruited from specialist outpatient clinics within the Queen Mary Hospital under the Hospital Authority of Hong Kong. Those who met the initial study criteria were referred by psychiatrists who ascertained their diagnosis of MDD with Structured Clinical Interview for Diagnostic Statistics Manual-5, Clinical Version (SCID-DSM-5, CV). Inclusion criteria included the following: age between 16 and 25 years, MDD as confirmed by SCID-DSM-5, a Hamilton Depression Rating Scale (HDRS) score of 14 or higher at screening and baseline (indicating at least mild or above in their severity of depressive symptoms), right-handedness, stable dosage of antidepressants or other depression treatments in the past four weeks, and the ability to read and write Chinese. Exclusion criteria included a history of significant head trauma; neurological disorders (e.g., epilepsy) or seizures; first-degree relatives with epilepsy or significant neurological disease; unstable medical conditions; comorbid disorders listed in DSM-5, e.g., schizophrenia, substance use disorder, intellectual disability etc.; pregnancy, lactating women, or women planning pregnancy; contraindications for imaging; and inability to provide informed consent.

### 2.3. Interventions

The treatment followed the tDCS protocol [35] commonly referred to upon considering safety and tolerability, comprising five consecutive sessions in five days of 30 min 2 mA tDCS. We utilised the Soterix Medical tDCS device (Soterix Medical, New York, NY, USA, Model 1 × 1 tDCS-CT) in the research lab and the Sooma Oy device (Sooma Depression Therapy Comfort, Helsinki, Finland) for patient self-administration. The use of the Sooma portable device facilitated decentralised administration, including a customised sham device for effective blinding. Blinding was ensured by assigning sham and active devices based on serial numbers, managed by an external data manager following a pre-determined randomisation sequence.

**Figure 3:**
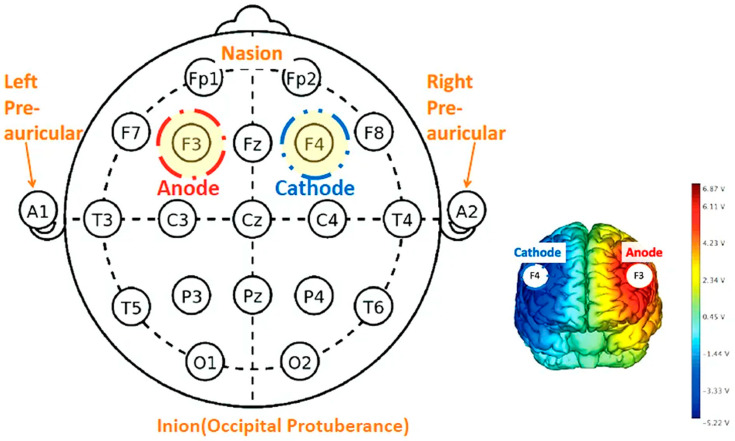
tDCS schematic diagram of electrode montage according to the 10–20 system.

Due to the limited availability of the Sooma devices, participants were randomly allocated to either the Sooma or Soterix device during their initial session based on availability. To ensure uniformity in tDCS delivery despite the use of two device types, we had standardisation procedures across sessions. Both devices operated under consistent settings to ensure uniformity in tDCS delivery. For all sessions, a current of 2 mA was applied with the anode placed over the left dorsolateral prefrontal cortex (DLPFC), corresponding to F3 in the international 10–20 EEG system, and the cathode placed over the right DLPFC (F4), as demonstrated in Figure 3. Saline-soaked sponge electrodes (5 cm × 7 cm; 35 cm^2^) were used, and the current was gradually ramped up and down during the first and last 30 s of each session to enhance comfort. For self-administered sessions, the researcher initially determined the appropriate cap size by measuring each participant’s head circumference during the intake session. Although the Soterix system is typically used in research settings and the Sooma system is designed for clinical/home use, before use, we verified that both systems delivered comparable stimulation protocols under the same parameters. Device allocation occurred only at the first supervised session, where a trained researcher ensured correct montage placement and calibration across both device types.

### 2.4. Procedures

Before the commencement of the study, participants were given an allocation number to receive either active tDCS or sham tDCS by an external data manager, who was not involved in the delivery of the trial. An independent researcher used a computer-generated list to make the random allocation number. Considering the challenges in subject recruitment and the feasibility of tDCS self-administration highlighted in our review, we designed the in-lab initiation phase which ensured participant safety, adherence, and protocol standardisation, while the remote self-administration phase minimised participant burden and improved convenience. The in-lab initiation phase was conducted at Queen Mary Hospital, where participants completed their first tDCS session under the supervision of trained investigators. This phase served as an orientation to ensure participants were familiarised with device operation, tolerability, and correct electrode placement. Parents of participants under the age of 18 also participated in the training session to provide additional support [36]. All participants underwent an initial in-person training to familiarize the use of tDCS, supplemented with instructional materials that use lay-language. For participants under the age 18, parents participated in the in-person training. Participants maintained a log to document the time and duration of each home-based session, with detailed checklists provided to ensure adherence to the intervention protocol. During the remote self-administration phase, participants completed four tDCS sessions at home using the Sooma portable device. Participants maintained a detailed log documenting the time and duration of each session and were provided with checklists to promote adherence. Four sessions were supervised by trained investigators in real time via videoconference to provide ongoing support. Daily contact was maintained to monitor treatment progress and encourage optimal compliance. The sessions were scheduled at least 24 h apart to address limitations identified in the previous RCT [37]. The unmasking of group allocation took place at the follow-up phase after all outcome variables had been collected. The USD 26 incentive was provided to the participants to subsidize the travel cost.

### 2.5. Blinding

To ensure blinding of the participant and their caregiver, at the beginning of the stimulation sessions, participants receiving both the active and sham stimulation would experience a ramp-up in current from 0 to 2 mA, and then the current would ramp down to 0 mA for participants receiving the sham stimulation [38]. Participants were told that they might experience sensations such as tingling, headache, or mild burning during the first 30–60 s of the stimulation session but that these sensations may subside afterwards as they became used to it. In other words, participants would not be able to tell if the diminishment of side effects was due to habituation (active tDCS) or the ramping down of the current (sham tDCS) [39].

### 2.6. Assessments and Outcomes

This study primarily aimed to assess the feasibility of conducting a tDCS RCT in youths with depression. Feasibility outcomes included recruitment success, data collection processes, session attendance, retention rates, randomisation procedures, and the feasibility of administering clinical and patient-reported outcome measures to identify suitable endpoints for future RCTs. These measures were evaluated to inform the design of larger-scale RCTs.

Baseline assessments (T0) included demographics, medical history, current medications, and depression severity. Post-intervention assessments (T1) were conducted to evaluate the feasibility of outcome measurement tools. The HDRS consists of 21 questions and the total score ranges from 0 (minimum) to 53 (maximum), with a higher score indicating more severe depressive symptoms [40]; it is used to explore the feasibility of assessing depressive symptoms in this population. The Chinese version of the Snaith–Hamilton Pleasure Scale (C-SHAPS) [41] and the Chinese version of the Dimensional Anhedonia Rating Scale (C-DARS) [42] were included to evaluate the multidimensional nature of depression and identify suitable measures for future trials. The C-SHAPS is a 14-item self-reported questionnaire where each item is scored from 0 (strongly disagree) to 3 (strongly agree), allowing for a total score range from 0 to 42. Higher scores on the C-SHAPS indicate more severe anhedonic symptoms, making it the gold standard for assessing anhedonia. Meanwhile, the C-DARS consists of 17 items, each ranging from 0 to 4, with total scores varying from 0 to 68. Here, higher scores represent lower levels of anhedonia. The C-DARS provides a comprehensive assessment of anhedonia across four domains: interest/pastimes, social interaction, sensory experience, and food/drink.

To explore the feasibility of monitoring potential adverse effects and tolerability, we adopted an Adverse Effects Questionnaire [21] and clinical observation of any unintended outcomes. Additionally, adherence was monitored by recording full participation in each tDCS session and noting any reasons for non-attendance. Knowledge of treatment conditions was assessed by asking participants at the end of the last stimulation session whether they believed they were receiving active tDCS stimulation and why. In addition, two Visual Analogue Scales (VASs) were used to measure the comfort score and the level of willingness to engage in the treatment. Each VAS contains a scale from 0 (minimum) to 10 (maximum), with higher scores indicating more comfort and stronger willingness to receive the treatment.

Given the possibility of tDCS with treatment-emergent mania, especially in youths (e.g., [26]), we included the Young Mania Rating Scale (YMRS) to evaluate the emergence of manic symptoms [43]. Functional outcomes were selected to assess the feasibility of evaluating a holistic understanding of treatment impacts, especially in a young demographic where social role and occupational functioning are key developmental milestones. The studies listed, such as NCT03897699 and ongoing trials like NCT06061653, focus on various functioning scales. The Social and Occupational Functioning Assessment Scale (SOFAS) was used as a rating scale measuring social and occupational functioning across work functioning, independent functioning, and immediate and extended social network functioning; the score ranges from 0 (minimum) to 100 (maximum), where a higher score indicates higher social and occupational functioning ability. The clinician-rated Global Functioning—Role Scale and Social Scale (GF-Role/-Social) assessed the role and social functioning ranges from 1 (minimum) to 10 (maximum), with a higher score indicating better social/role functioning [44]. The Role Functioning Scale (RFS) was used to measure role functioning in four areas: work productivity, independent living, and immediate and extended social network relationships [45]. The score ranges from 0 (minimum) to 7 (maximum) on each aspect, with a higher score indicating better role functioning.

### 2.7. Statistical Analysis

The statistical analysis for this pilot feasibility study was primarily descriptive and exploratory due to the small sample size (*n* = 8). Based on a pooled analysis showing a mean HDRS score reduction of −9.6 (SD ≈ 6.25), the estimated effect size (Cohen’s d) is approximately 1.54—a large effect. A fully powered RCT would require at least 21 participants per arm (α = 0.05, power = 80%). However, as this pilot study focuses on feasibility and preliminary signals of efficacy, we adopted a pragmatic approach and enrolled 8 participants. While not powered for hypothesis testing, this sample provides valuable data to guide the design of a future, larger trial. Baseline demographic and clinical characteristics, including age, sex, education level, time since depression onset, and baseline HDRS scores, were summarised using means and standard deviations for continuous variables and counts and percentages for categorical variables. Feasibility outcomes, including adherence, adverse effects, and blinding success, were analyzed descriptively. Primary and secondary clinical outcomes, such as changes in HDRS, SHAPS, C-DARS, and SOFAS scores, were reported as mean changes from baseline to post-intervention within each group. Fisher’s exact test was used for categorical variables and Mann–Whitney U tests were used for comparing continuous variables. Formal hypothesis testing was avoided due to the exploratory nature of this pilot study, and effect sizes (Cohen’s d) were calculated to estimate the magnitude of observed differences between groups. A two-tailed p-value of less than 0.05 was used as a reference for exploratory analyses. All computations were performed using R version 4.3.2 for MacOS and Microsoft Excel.

## 3. Results

Eight participants (five males, and three females, aged 16–24 years) were successfully recruited and retained, with no dropouts or technical issues throughout the study. All participants completed their assigned sessions, achieving 100% adherence, and the feasibility of randomisation, outcome measurement, and session attendance was confirmed (Table 2).

### 3.1. Primary Feasibility Outcomes

Recruitment and retention were successful, with all participants engaging in the study from baseline to post-intervention. Data collection for clinical, and functional assessments was feasible, with no missing data reported. Randomisation procedures were effective, and blinding was maintained, as participants in both groups were equally likely to believe they had received active tDCS stimulation.

### 3.2. Exploratory Outcomes

As summarised in Table 3, exploratory analysis of clinical outcomes suggested reductions in HDRS scores in both groups, while the very small sample size prevented any efficacy conclusions. The active tDCS group showed a greater mean reduction in HDRS scores (−4.75; CI: −6.27 to −3.23) compared to the sham group (−3.75; C: −2.26 to 9.76); however, this difference was not statistically significant (*p* = 0.48). The active tDCS group had a significantly higher HDRS score at baseline than the sham group. We conducted a sensitivity analysis with baseline scores as covariates. After adjustment, there remained no significant difference in the reduction in HDRS score (*F*(1,6) = 0.91, *p* = 0.385), confirming that our result was independent of baseline severity. Secondary measures, including anhedonia (C-SHAPS and C-DARS), functional scales (SOFAS, RFS), and mania severity (YMRS), were successfully administered, confirming the feasibility of measuring multidimensional outcomes in this population. While no significant changes were observed, the active group demonstrated a small, non-significant improvement in functioning and anhedonia scores compared to the sham group.

### 3.3. Tolerability and Engagement

Table 4 summarizes the tolerability and compliance for both active and sham tDCS groups, with a sample size of four each. The active group reported adverse effects such as tingling (0.10%) and headaches (0.15%), whereas the sham group experienced no adverse effects. Compliance was at 100% in both groups. When comparing in-person-delivered versus self-administered tDCS modalities, both settings reported similar minor adverse effects (tingling and headaches) with equally high compliance rates. On day 5, prior to unblinding and after completing all assessments, participants were asked to guess whether they received ‘active’ or ‘sham’ tDCS. Both groups have a similar % of subjects who perceived they had received the real intervention. None had prior experience with tDCS, yet all believed they were receiving the active treatment. Their belief stemmed mainly from experiencing a tactile sensation they associated with electrical stimulation, described as a “mild buzzing” on the scalp during the sessions. Additionally, some participants noted feeling calmer and improved overall, which further contributed to their perception of receiving active treatment.

At baseline, the sham group exhibited stronger willingness to participate in the tDCS treatment (M = 8.50; SD = 0.71) and higher comfort (M = 9.00; SD = 0.00) scores compared to the active group (willingness to participate: M = 6.00; SD = 0.82; comfort: M = 7.25; SD = 0.96). Both groups demonstrated an increase in the score reflecting their willingness to participate; there was no significant difference in the comfort score.

## 4. Discussion

Our review indicated the preliminary efficacy of tDCS for youth depression as an augmentative treatment—a demographic with notably few treatment options. The reviewed studies, however, highlight recruitment challenges and a high risk of bias. To address these gaps, this pilot study offers preliminary feasibility data for implementing review-informed tDCS parameters (2 mA, 30 min sessions, DLPFC targeting) in real-world youth psychiatric settings. The pilot trial’s decentralised elements may address accessibility bias.

Three major findings emerged: first, we found preliminary evidence supporting the feasibility and acceptability of conducting a decentralised tDCS RCT in youths, and the protocol of bifrontal montage demonstrated a favourable effect on reducing depression severity, as evidenced by improvements in depression scores. Differences between the active and sham groups were not statistically significant, likely due to the small sample size and exploratory nature of the study, but the directionality supports further inquiry in better-powered trials. Second, our study highlighted the feasibility of a decentralised tDCS protocol involving self-administration for youths. The compliance rate, willingness to participate, and comfort score were encouraging. Third, no unexpected outcomes or severe adverse effects were reported throughout the trial, consistent with reviewed tDCS studies, and the simulations were well tolerated by the participants. The absence of severe adverse events further supports the safety of tDCS in this population. Taken together, our review and pilot trial provide preliminary observations on participant engagement, protocol adherence, and procedural logistics, which may inform future studies and clinical implementation efforts in digital mental health settings.

The implementation of a hybrid decentralised model (5-session DLPFC tDCS at 2 mA with caregiver support) suggests that self-administered neuromodulation may be feasible in youths, maintaining >80% adherence rates and addressing practical barriers in this demographic. Despite the common issue of skipping daily neuromodulation sessions in clinical groups with depression [46], no specific predictors were identified for non-adherence [34]. Contributing factors to missed sessions typically included symptoms of anhedonia and low motivation [47], the side effects of medication, the nature of the psychiatrist–patient relationship [48], and the limited availability of our clinical centre during standard office hours (9 AM to 5:40 PM). Most participants in our study were students with daytime commitments, yet they were given the flexibility to select a one-week period that best fit their schedules. This approach resulted in no dropouts or losses in follow-up, although the rescheduling of several sessions was necessary. Sessions were often moved to evening hours to accommodate participants’ regular working or schooling commitments, due to difficulty in heading out from home for treatment, or due to self-reported low energy levels in a day. Nonetheless, they attended the session later in the evening after a reminder message and further accommodation of the treatment schedule beyond office hours.

Recent studies on self-administered tDCS in psychiatric populations have shown positive evidence of its safety [37,49], tolerability [50,51], and efficacy [49]. In our study, low adherence and usability issues, common in telehealth interventions for depression [52], were addressed through enhanced supervision between tDCS supervisors and users. Most of our participants had additional support from lay assistants (i.e., caregivers or family members serving as co-participants in the research study [53]. Training, videoconferencing, and logging of tolerability were employed to ensure safe and responsible use [36]. The current strength of 2 mA appeared to be as tolerable as sham tDCS stimulation in this sample [20]. Additionally, the blinding of active versus sham conditions suggests that our study design was adequate for our participants who had not previously received tDCS [54,55]. Our study demonstrated the feasibility of conducting a trial of tDCS in youth depression.

In line with the meta-analytic evidence that active tDCS was associated with greater improvements in depressive symptoms [56,57,58], our trial employed a comparable tDCS montage to assess clinical effects. Research indicates that tDCS is particularly effective in individuals with MDD who are not treatment-refractory [59]. A recent study demonstrating significant improvement in depression treatment specifically excluded participants who had failed two or more antidepressant trials [49], suggesting a correlation between treatment refractoriness and the diminished clinical efficacy of tDCS [60]. The youth demographic, characterised by typically shorter durations of illness and younger ages at onset, may be especially responsive to tDCS [61]. Conventional treatments for youth depression, such as antidepressants and psychotherapy, often fall short [5,6], with many youths not responding adequately and treatment adherence being generally low due to side effects and other barriers [11]. This underscores the significance of minimally burdensome interventions like tDCS, which also aligns with the limited but favourable evidence of clinical efficacy from our systematic review of tDCS for youth depression.

Our study applied anodal tDCS to the left DLPFC, targeting shared prefrontal–amygdala network dysfunction observed in both adults and youths [62,63], consistent with the frontal imbalance hypothesis of depression [64]. However, physiological variations in response to tDCS between younger persons and adults—including the scalp-to-brain distance, thickness of the scalp and calvarium, cerebrospinal fluid volume, and developmental changes in tissue architectures—should be considered [65,66,67]. Using a computational modelling approach, applying a lower current intensity (1 mA) may achieve brain current densities, in those under 12 years of age, that are comparable, on average, to the densities seen in adults with a 2 mA current [68]. To address these age-dependent physiological differences and optimize tDCS parameters, the integration of tDCS with neuroimaging techniques like fMRI and computational modelling approaches may further help to elucidate age-dependent effects on the developing brain [30]. This is particularly pertinent in the youth, where developmental trajectories of brain maturation may affect the response to neuromodulatory interventions. The developing brain’s shifting balance of excitation–inhibition might offer a unique window in which tDCS might modulate neural circuits involved in depression [69].

Our findings provide positive evidence regarding the tolerability and safety profile of tDCS in treating youth depression. All participants experienced a side effect profile comparable to that observed in adult populations, primarily reporting mild headaches and no severe adverse effects, aligning with the adult tDCS literature, which highlights its safety and that it has minimal and manageable side effects [21,60]. It is noteworthy that existing RCTs with robust study designs focusing on youths with mental disorders have predominantly examined neurodevelopmental disorders. Specifically, five RCTs have investigated autism spectrum disorders with treatments ranging from 5 to 15 sessions of 1–1.5 mA targeting the left DLPFC [66,70,71,72,73] and two studies have addressed attention deficit hyperactivity disorder with 5–12 sessions of 0.75–1.5 mA on the left DLPFC [74,75]. Positive evidence on safety and tolerability was consistently reported. Nonetheless, the application of tDCS in the developing brain may produce potentially unintended or adverse effects. Theoretically, the developing brain has a shifted balance of excitation–inhibition, resting closer to the seizure threshold [76]. Yet, there have been no reports of seizures from a systematic review of 384 children undergoing neuromodulation [20]. It is also common for young participants who have a history of seizure or underlying neurological or brain insults to undergo tDCS for the potential treatment effect [77,78]. Importantly, we must consider the risk of treatment-emergent mania in tDCS use in youths [79]. There was a report of treatment-emergent hypomania in a 33-year-old individual with bipolar II disorder, already on mood stabilizers, during their second course of tDCS using fronto-extracephalic stimulation, as opposed to their first course using bifrontal stimulation [80]. In our study with a brief protocol of five-session tDCS, we observed no change in YMRS scores pre- and post-intervention. Practically, it is also important to consider unanticipated findings (albeit positive, rather than adverse), as tolerability refers to the presence of uncomfortable and unintended effects without structural or functional damage [81]. For instance, a feasibility study on tic disorder and epilepsy reported that a child aged 12 had a marked reduction in tic severity while tDCS aimed to reduce autism symptoms [82]. This suggests that due to the developing brain’s unique characteristics, standard tDCS montages might lead to indirect stimulation at non-targeted sites, potentially modulating symptoms in a clinically meaningful manner. Unintended effects should be assessed in further research on tDCS in youths, alongside their relatively established safety profiles. Furthermore, the interpretation of tDCS outcomes should be closely tied to clearly defined therapeutic endpoints and temporally relevant outcome measures. In our study, while short-term effects were assessed immediately pre- and post-intervention, longer-term effects or delayed responses were not captured. Given that the developing brain may respond to neuromodulation over variable timeframes, future research should incorporate multiple follow-up time points to map the trajectory and durability of both intended and unintended effects.

Our study had some methodological limitations. First, a key strength of this review was the inclusion of non-English publications, ongoing trials, and case reports to synthesize the full spectrum of available evidence. However, this inclusive approach also introduced the possibility of incorporating studies with a higher risk of bias and lower methodological quality. Although this strategy allowed us to address the limited availability of high-quality studies in this emerging field, it may have increased the overall risk of bias in the findings. Second, even though the depression scores showed improvements, the small sample warrants caution while making any conclusions. We had a low-intensity protocol. Only two assessments were conducted in the trial, with the last occurring immediately at the end of treatment (day 5). This design does not address carry-over effects, leaving the durability of treatment outcomes unexamined. Nonetheless, our study aimed to investigate the feasibility instead of the efficacy of tDCS treatment in youth depression. Third, while the hybrid design retained essential in-person components such as training and the initial session, the decentralised administration of the remaining sessions may have introduced heterogeneity when compared to a fully in-person approach. This potential limitation underscores the importance of examining whether the hybrid model influences treatment outcomes compared to a centralised protocol. Future research should investigate the effect of the intervention delivery mode on consistency and efficacy, especially in decentralised clinical trials (DCTs).

## 5. Conclusions

In conclusion, our review and feasibility study underscore the potential of tDCS as a viable and feasible treatment option for youth depression, a domain where its applications remain notably sparse. The first part of our study revealed a significant lacuna in the literature review, the heterogeneity of tDCS stimulation paradigms, and the high risk of bias in the existing literature. The second part of our study demonstrated that conducting a sham-controlled tDCS RCT for youth depression is feasible, with high retention rates, adequate adherence, and successful implementation of blinding procedures.

Our study offers initial observations of the existing evidence and insights into the feasibility of applying review-informed tDCS parameters (2 mA, 30 min sessions, DLPFC targeting) in real-world youth psychiatric settings. The observed retention rates, maintenance of blinding, and trend toward symptom improvement suggest the plausibility of further investigation. While the pilot was not designed or powered to assess efficacy, the research might be better conceptualised as providing preliminary groundwork for future investigations that could more rigorously assess therapeutic potential. It is also worth noting that tDCS may modulate neuroplasticity, which could be particularly relevant for early-stage depression in this demographic, where pharmacological or other neuromodulatory interventions are poorly tolerated. Further RCTs in this field are necessary for advancing the development and refinement of early intervention strategies that halt disease progression in this vulnerable group.

## Figures and Tables

**Table 1:** Summary of eligible articles and unpublished clinical trials using tDCS in youths with depression.

**1.** **Clinical Trial**								
Name of First Author/Publication Year	Design	N	Diagnosis	Age Range (N)	Control	Anode/Cathode	Protocol	POM
Baibujiapu et al., 2017 [24]	Sham-Controlled Study ^(a)^	Active = 32 Sham = 33	Major Depressive Disorder	10–17	Sham-tDCS + Sandplay Therapy	Primary somatosensory cortex/contralateral shoulder	15 sessions; 1.2 mA; 20 min	HDRS at (pre: 28.7 ± 4.3; post: 20.5 ± 3.6) (*p* < 0.001 between groups) ^(b)^
Zhang et al., 2023 [25]	Open-label Study	28	Major Depressive Episode	14–17	-	HD-tDCS Central anode: L-DLPFC	20 sessions; 2 mA; 20 min	HDRS-17 (pre: 24.14 ± 5.79; post: 13.14 ± 5.49; *t* = −11.383, *p* < 0.001) ^(b)^
**2.** **Case Reports**								
Name of First Author/Publication Year	Design	N	Diagnosis	Age (N)	Control	Anode/Cathode	Protocol	POM
Baliga et al., 2020 [26]	Case Report	1	Bipolar Depression	21	-	L-DLPFC/R-DLPFC	10 sessions; 2 mA; 30 min	HDRS (reduction from 20 to 9) (30%)
Clayton et al., 2018 [27]	Case Report	1	Major Depression with Multiple Sclerosis	19	-	L-DLPFC	2 sessions; 1.5 mA; 20 min ^(c)^	BDI (reduction from 12 to 0)
Shankar et al., 2023 [28]	Case Report	1	Treatment-resistant depression	16	-	Not reported	20 sessions; n-r; after ketamine infusion	HDRS-17 (reduction from 15 to 8)
Sreeraj, et al., 2016 [29].	Case Report	1	Depression with pregnancy	23	-	L-DLPFC/R-DLPFC	10 sessions; 2 mA; 30 min	HDRS (reduction from 18 to 6), HAMA
**3.** **Ongoing unpublished clinical trials**								
Trial ID (Status)	Design	N	Diagnosis	Age range (N)	Control	Anode/Cathode	Protocol	POM
ChiCTR2000039503 (ongoing)	Case–Control Observation Study	50	Major Depressive Disorder	10–22	-	Not reported	Not reported	ASIQ, BSI, BRMS, CGI, CTQ, HAMA, HDRS, MPQ-SF, PSQI, SHAPS, SDSS
ChiCTR2400079464 (ongoing)	Double-blind RCT	40	Major Depressive Disorder	13–17	Sham tDCS	Not reported	Not reported	HDRS-17
DRKS00027066 (ongoing)	Double-blind RCT	100	Major Depressive episode	13–17	Sham tDCS	L-DLPFC/R-DLPFC	10 sessions; 2 mA	BDI-II
NCT03368469 (withdrawn) ^(d)^	Open-label, Single-arm Study	20 ^(e)^	Epilepsy and Depressive Disorder	10–21	Sham tDCS	lDPFC/R-SOA	10 sessions; 2 mA; 20 min	CDRS-R
NCT03897699 (completed)	Quadruple-blind RCT	68 (Actual: 36)	Major Depressive Disorder (girls only)	17–24 (mean age 20.2)	Sham tDCS + Mindful Breathing Training	L-DLPFC/R-DLPFC	10 sessions; 2 mA; 20 min	Change in DLPFC Connectivity; Amygdala/DMN Secondary outcome measure: MARDS: Active group 6.47 (4.69); Control Group 5.95 (4.04) ^(f)^ Adverse Effects:
NCT04780152 (ongoing)	Double-blind RCT	172	Major Depressive Disorder	10–17	Sham tDCS + fluoxetine	L-DLPFC/R-DLPFC	10 sessions; 2 mA; 30 min	CDI
NCT05498441 (ongoing)	Double-blind RCT	120	Major Depressive Episode	13–18	Routine HD-tDCS	Central anode: personalised HD-tDCS with 4 × 1 ring montage on L- DLPFC	20 sessions; 20 min	HDRS-17, RBANS, MRI and DTI imaging
NCT06061653 (ongoing)	Double-blind RCT	60	Major Depressive Disorder	12–18	IPT + HD-tDCS	Not reported	Not reported	HDRS-24; CRDS-R

ASIQ, Adolescent Self Injury Questionnaire; BDI, Beck Depression Inventory; BRMS, Bech–Rafaelsan Mania Rating Scale; BSI, Beck Suicidal Ideation Scale; CDI, Children Depression Inventory; CDRS, Children’s Depression Rating Scale; CDRS, Children’s Depression Rating Scale- Revised; CGI, Clinical Global Impression Scale; CTQ, Childhood Trauma Questionnaire; DMN, default mode network; DTI, diffusion tensor imaging; HAMA, Hamilton Anxiety Rating Scale; HDRS, Hamilton Depression Rating Scale; HD-tDCS, high-definition transcranial direct current stimulation; MADRS. Montgomery–Asberg Depression Rating Scale; MDD, major depressive disorder; IPT, Interpersonal Therapy; lDLPFC, left dorsolateral prefrontal cortex; MPQ-SF, McGill Pain Scale; MRI, magnetic resonance imaging; POMs, primary outcome measures; PSQI, Pittsburgh Sleep Quality Index; SDSS, Signs of Depression Screening Scale; SHAPS, Snaith Hamilton Pleasure Rating Scale; SPA: supraorbital area; RBANS, Repeatable Battery for the Assessment of Neuropsychological Status test ^(a)^ The randomisation or blinding was not reported. ^(b)^ No adverse effect was assessed or reported. The study was not registered. ^(c)^ Home-based tDCS (under remote supervision). ^(d)^ The study team was unable to recruit and enrol subjects. The study is now closed. ^(e)^ Anticipated. ^(f)^ Adverse effects: no serious adverse events; the 3 most common adverse effects are: sleepiness (71.43%), itchiness (66.67%) and unusual feelings on the skin of the head (52.38%).

**Table 2:** Participants’ characteristics.

	Sex/Age	Education (Years)	Time Since Depression (Weeks)	HDRS Severity (Baseline)	Device Assigned	Treatment Completion	Self-Administration Compliance	Adverse Effects	Blinding Guess
1.	Male/16	8	104	Mild (14)	Sooma (Home-Based)	5 sessions completed without issues	Completed 5/5 home sessions, no issues	None	Not correct
2.	Male/20	13	156	Severe (21)	Soterix (Hospital-Based)	5 sessions completed (1 session rescheduled)	-	Tingling (Mild)	Correct
3.	Female/20	13	312	Mild (14)	Sooma (Home-Based)	5 sessions completed, with prompting	Completed 5/5 home sessions, 1 missed, prompted the same day for makeup session	None	Not correct
4.	Female/17	10	-	Severe (20)	Sooma (Home-Based)	5 sessions completed without issues	Completed 5/5 home sessions, no issues	Tingling (Mild)	Correct
5.	Male/16	11	260	Moderate (18)	Soterix (Hospital-Based)	5 sessions completed (2 sessions rescheduled)	-	None	Not correct
6.	Male/21	15	312	Moderate (15)	Sooma (Home-Based)	5 sessions completed (minor delays)	Completed 5/5 home sessions, minor delays	None	Correct
7.	Male/20	13	52	Moderate (15)	Soterix (Hospital-Based)	5 sessions	-	None	Not correct
8.	Female/24	15	312	Moderate (15)	Soterix (Hospital-Based)	5 sessions (rescheduling required)	-	Tingling, Headache (Mild)	Correct

**Table 3:** Report of mean changes in pre-post tDCS treatments.

Score/Group	T0 Mean	(S.D.)	T1 Mean	(S.D.)	Mean Difference	(S.D.)	t	*p*	d
HDRS: *p* = 1									
Active tDCS	18.50	(2.65)	13.75	(2.06)	−4.75	(0.96)	−0.72	0.48	−0.32
Sham tDCS	13.75	(1.50)	10.00	(2.45)	−3.75	(3.78)			
SHAPS: *p* = 1									
Active tDCS	34.25	(4.50)	37.00	(4.83)	2.75	(4.20)	1.23	0.236	0.55
Sham tDCS	45.50	(3.32)	46.00	(3.56)	0.50	(0.57)			
C-DARS: *p* = 1									
Active tDCS	24.00	(8.17)	27.00	(7.07)	3.00	(7.96)	1.03	0.32	0.46
Sham tDCS	55.50	(10.60)	53.00	(7.39)	−2.50	(7.77)			
YMRS: N.A.									
Active tDCS	3.00	(0.40)	3.00	(0.40)	-	-	N.A.		
Sham tDCS	2.80	(0.54)	2.80	(0.54)	-	-			
SOFAS: *p* = 0.429									
Active tDCS	6.00	(0.82)	6.75	(1.50)	0.75	(0.96)	0.65	0.52	0.29
Sham tDCS	8.50	(0.71)	9.50	(0.01)	1	(0.56)			
VAS—Comfort: *p* = 1									
Active tDCS	7.25	(0.96)	6.75	(1.71)	−0.50	(1.29)	−1.42	0.26	−0.78
Sham tDCS	9.00	(0.00)	10.00	(0.00)	1	(0.00)			

T0, Baseline; T1, post-intervention; HDRS, Hamilton Depression Rating Scale; C-SHAPS, Chinese version of the Snaith–Hamilton Pleasure Scale; C-DARS, Chinese version of the Dimensional Anhedonia Rating Scale; YMRS, Young Mania Rating Scale; VAS, Visual Analogue Scale.

**Table 4:** Comparative table of assessment of tolerability and compliance across treatment conditions and modes.

	Active Group (*n* = 4)		Sham Group (*n* = 4)	
Adverse Effects	Frequency	%	Frequency	%
Tingling	2	0.10%	0	
Skin redness	0		0	
Burning sensation	0		0	
Headache	3	0.15%	0	
Fatigue	0		0	
Itching	0		0	
Scalp pain	0		0	
Neck pain	0		0	
Sleepiness	0		0	
Trouble concentrating	0		0	
Acute mood change	0		0	
Compliance		100%		100%
	Hospital-Based (*n* = 4)		Home-Based (*n* = 4)	
Adverse Effects	Frequency	%	Frequency	%
Tingling	1	0.05%	1	0.05%
Skin redness	0		0	
Burning sensation	0		0	
Headache	2	0.10%	1	0.05%
Fatigue	0		0	
Itching	0		0	
Scalp pain	0		0	
Neck pain	0		0	
Sleepiness	0		0	
Trouble concentrating	0		0	
Acute mood change	0		0	
Compliance		100%		100%

## Data Availability

The data that support the findings of this study are available from the corresponding author upon reasonable request.

## Supplementary Materials

The following supporting information can be downloaded at https://www.mdpi.com/article/10.3390/jcm14093152/s1: Table S1: Items that should be included in a PRISMA statement; Table S2: Risk of bias assessment; Table S3: Items that should be included in a CONSORT statement; Table S4: Subgroup analysis of home-based vs. hospital-based tDCS.
