# Supplementary material for: Transcranial Direct Current Stimulation (tDCS) in the Treatment of Youth Depression: Integrating Literature Review Insights in a Pilot Clinical Trial
Source: J Clin Med. 2025 May 1;14(9):3152. doi: 10.3390/jcm14093152 (PMC12072900; doi:10.3390/jcm14093152)
Supplement: Supplementary file 1 [file jcm-14-03152-s001.zip › jcm-3581204-supplementary.pdf]

Supplementary Materials

Supplementary Table S1. Items that should be included in PRISMA Statement

Supplementary Table S2. Risk of bias assessment

Supplementary Table S3. Items that should be included in CONSORT Statement

Supplementary Table S4. Variability in device use (Sooma vs Soterix) and administration context (home vs lab) should be presented in subgroup analysis.

Section and Topic	Item #	Checklist item	Location where item is reported
**TITLE**			
Title	1	Identify the report as a systematic review.	1
**ABSTRACT**			
Abstract	2	See the PRISMA 2020 for Abstracts checklist.	2
**INTRODUCTION**			
Rationale	3	Describe the rationale for the review in the context of existing knowledge.	4-5
Objectives	4	Provide an explicit statement of the objective(s) or question(s) the review addresses.	5
**METHODS**			
Eligibility criteria	5	Specify the inclusion and exclusion criteria for the review and how studies were grouped for the syntheses.	5
Information sources	6	Specify all databases, registers, websites, organisations, reference lists and other sources searched or consulted to identify studies. Specify the date when each source was last searched or consulted.	5
Search strategy	7	Present the full search strategies for all databases, registers and websites, including any filters and limits used.	5
Selection process	8	Specify the methods used to decide whether a study met the inclusion criteria of the review, including how many reviewers screened each record and each report retrieved, whether they worked independently, and if applicable, details of automation tools used in the process.	5
Data collection process	9	Specify the methods used to collect data from reports, including how many reviewers collected data from each report, whether they worked independently, any processes for obtaining or confirming data from study investigators, and if applicable, details of automation tools used in the process.	5
Data items	10a	List and define all outcomes for which data were sought. Specify whether all results that were compatible with each outcome domain in each study were sought (e.g., for all measures, time points, analyses), and if not, the methods used to decide which results to collect.	5
	10b	List and define all other variables for which data were sought (e.g., participant and intervention characteristics, funding sources). Describe any assumptions made about any missing or unclear information.	5-6
Study risk of bias assessment	11	Specify the methods used to assess risk of bias in the included studies, including details of the tool(s) used, how many reviewers assessed each study and whether they worked independently, and if applicable, details of automation tools used in the process.	6, S2_Risk of bias assessment
Effect measures	12	Specify for each outcome the effect measure(s) (e.g., risk ratio, mean difference) used in the synthesis or presentation of results.	6
Synthesis methods	13a	Describe the processes used to decide which studies were eligible for each synthesis (e.g., tabulating the study intervention characteristics and comparing against the planned groups for each synthesis (item #5)).	5-6, Table 1
	13b	Describe any methods required to prepare the data for presentation or synthesis, such as handling of missing summary statistics, or data conversions.	5-6
	13c	Describe any methods used to tabulate or visually display results of individual studies and syntheses.	5-6, Table 1
	13d	Describe any methods used to synthesize results and provide a rationale for the choice(s). If meta-analysis was performed, describe the model(s), method(s) to identify the presence and extent of statistical heterogeneity, and software package(s) used.	6
	13e	Describe any methods used to explore possible causes of heterogeneity among study results (e.g., subgroup analysis, meta-regression).	NA
	13f	Describe any sensitivity analyses conducted to assess robustness of the synthesized results.	NA
Reporting bias assessment	14	Describe any methods used to assess risk of bias due to missing results in a synthesis (arising from reporting biases).	S2_Risk of bias assessment

Section and Topic	Item #	Checklist item	Location where item is reported
Certainty assessment	15	Describe any methods used to assess certainty (or confidence) in the body of evidence for an outcome.	6
**RESULTS**			
Study selection	16a	Describe the results of the search and selection process, from the number of records identified in the search to the number of studies included in the review, ideally using a flow diagram.	Fig.1
	16b	Cite studies that might appear to meet the inclusion criteria, but which were excluded, and explain why they were excluded.	Fig.1
Study characteristics	17	Cite each included study and present its characteristics.	Table 2
Risk of bias in studies	18	Present assessments of risk of bias for each included study.	S2_Risk of bias assessment
Results of individual studies	19	For all outcomes, present, for each study: (a) summary statistics for each group (where appropriate) and (b) an effect estimate and its precision (e.g., confidence/credible interval), ideally using structured tables or plots.	Table 3
Results of syntheses	20a	For each synthesis, briefly summarise the characteristics and . 4 among contributing studies.	NA
	20b	Present results of all statistical syntheses conducted. If meta-analysis was done, present for each the summary estimate and its precision (e.g., confidence/credible interval) and measures of statistical heterogeneity. If comparing groups, describe the direction of the effect.	NA
	20c	Present results of all investigations of possible causes of heterogeneity among study results.	NA
	20d	Present results of all sensitivity analyses conducted to assess the robustness of the synthesized results.	NA
Reporting biases	21	Present assessments of risk of bias due to missing results (arising from reporting biases) for each synthesis assessed.	NA
Certainty of evidence	22	Present assessments of certainty (or confidence) in the body of evidence for each outcome assessed.	NA
**DISCUSSION**			
Discussion	23a	Provide a general interpretation of the results in the context of other evidence.	6-7
	23b	Discuss any limitations of the evidence included in the review.	5-6
	23c	Discuss any limitations of the review processes used.	6-7
	23d	Discuss implications of the results for practice, policy, and future research.	6-7
**OTHER INFORMATION**			
Registration and protocol	24a	Provide registration information for the review, including register name and registration number, or state that the review was not registered.	4
	24b	Indicate where the review protocol can be accessed, or state that a protocol was not prepared.	4 & S1_Prospero
	24c	Describe and explain any amendments to information provided at registration or in the protocol.	NA
Support	25	Describe sources of financial or non-financial support for the review, and the role of the funders or sponsors in the review.	NA
Competing interests	26	Declare any competing interests of review authors.	14-15
Availability of data, code and	27	Report which of the following are publicly available and where they can be found: template data collection forms; data extracted from included studies; data used for all analyses; analytic code; any other materials used in the review.	14-15

Section and Topic	Item #	Checklist item	Location where item is reported
other materials			

From: Page MJ, McKenzie JE, Bossuyt PM, Boutron I, Hoffmann TC, Mulrow CD, et al. The PRISMA 2020 statement: an updated guideline for reporting systematic reviews. BMJ 2021;372:n71. doi: 10.1136/bmj.n71.
This work is licensed under CC BY 4.0. To view a copy of this license, visit https://creativecommons.org/licenses/by/4.0/

Supplementary Table S1. Items that should be included in PRISMA Statement

Study	D1	D2	D3	D4	D5	Overall
**Baibujiapu et al., 2017**	-	-	!	-	!	-
**Zhang et al., 2023**	-	-	!	-	!	-

Domains:
D1: Bias arising from the randomisation process.
D2: Bias due to deviations from intended intervention.
D3: Bias due to missing outcome data.
D4: Bias in measurement of the outcome.
D5: Bias in selection of the reported result.

Judgement

-	High
!	Some concerns
+	Low

Supplementary Table S2. Risk of bias assessment

CONSORT 2010 checklist of information to include when reporting a pilot or feasibility trial*

Section/Topic	Item No	Checklist item	Reported on page No
**Title and abstract**			
	1a	Identification as a pilot or feasibility randomised trial in the title	1
	1b	Structured summary of pilot trial design, methods, results, and conclusions (for specific guidance see CONSORT abstract extension for pilot trials)	2
**Introduction**			
Background and objectives	2a	Scientific background and explanation of rationale for future definitive trial, and reasons for randomised pilot trial	3-7
	2b	Specific objectives or research questions for pilot trial	7
**Methods**			
Trial design	3a	Description of pilot trial design (such as parallel, factorial) including allocation ratio	7
	3b	Important changes to methods after pilot trial commencement (such as eligibility criteria), with reasons	NA
Participants	4a	Eligibility criteria for participants	7-8
	4b	Settings and locations where the data were collected	8
	4c	How participants were identified and consented	8
Interventions	5	The interventions for each group with sufficient details to allow replication, including how and when they were actually administered	8-9

Outcomes	6a	Completely defined prespecified assessments or measurements to address each pilot trial objective specified in 2b, including how and when they were assessed	9
	6b	Any changes to pilot trial assessments or measurements after the pilot trial commenced, with reasons	NA
	6c	If applicable, prespecified criteria used to judge whether, or how, to proceed with future definitive trial	NA
Sample size	7a	Rationale for numbers in the pilot trial	10
	7b	When applicable, explanation of any interim analyses and stopping guidelines	NA
Randomisation:			
Sequence generation	8a	Method used to generate the random allocation sequence	8
	8b	Type of randomisation(s); details of any restriction (such as blocking and block size)	8
Allocation concealment mechanism	9	Mechanism used to implement the random allocation sequence (such as sequentially numbered containers), describing any steps taken to conceal the sequence until interventions were assigned	9
Implementation	10	Who generated the random allocation sequence, who enrolled participants, and who assigned participants to interventions	9
Blinding	11a	If done, who was blinded after assignment to interventions (for example, participants, care providers, those assessing outcomes) and how	9
	11b	If relevant, description of the similarity of interventions	9
Statistical methods	12	Methods used to address each pilot trial objective whether qualitative or quantitative	9-10
**Results**			

Participant flow (a diagram is strongly recommended)	13a	For each group, the numbers of participants who were approached and/or assessed for eligibility, randomly assigned, received intended treatment, and were assessed for each objective	p.11, Fig.1
	13b	For each group, losses and exclusions after randomisation, together with reasons	p.11, Fig.1
Recruitment	14a	Dates defining the periods of recruitment and follow-up	8
	14b	Why the pilot trial ended or was stopped	NA
Baseline data	15	A table showing baseline demographic and clinical characteristics for each group	Tables 2 & 3
Numbers analysed	16	For each objective, number of participants (denominator) included in each analysis. If relevant, these numbers<br>should be by randomised group	Tables 2 & 3 & 4
Outcomes and estimation	17	For each objective, results including expressions of uncertainty (such as 95% confidence interval) for any<br>estimates. If relevant, these results should be by randomised group	p.11,Tables 3 & 4
Ancillary analyses	18	Results of any other analyses performed that could be used to inform the future definitive trial	p.11, Tables 2 & 3 & 4
Harms	19	All important harms or unintended effects in each group (for specific guidance see CONSORT for harms)	p.11. Table 4
	19a	If relevant, other important unintended consequences	NA
**Discussion**			
Limitations	20	Pilot trial limitations, addressing sources of potential bias and remaining uncertainty about feasibility	13

Generalisability	21	Generalisability (applicability) of pilot trial methods and findings to future definitive trial and other studies	13
Interpretation	22	Interpretation consistent with pilot trial objectives and findings, balancing potential benefits and harms, and considering other relevant evidence	11-14
	22a	Implications for progression from pilot to future definitive trial, including any proposed amendment	NA
**Other information**			
Registration	23	Registration number for pilot trial and name of trial registry	2,14
Protocol	24	Where the pilot trial protocol can be accessed, if available	Clinical Trials.gov
Funding	25	Sources of funding and other support (such as supply of drugs), role of funders	14-15
	26	Ethical approval or approval by research review committee, confirmed with reference number	IRB UW24-035

Supplementary Table S3. Items that should be included in CONSORT Statement

Supplementary Table S4. Variability in device use (Sooma vs Soterix) and administration context (home vs lab) should be presented in subgroup analysis.

Results of t-test analysis by treatment mode								
	**Hospital-based tDCS (*n* = 4)**				**Home-based tDCS (*n* = 4)**			
	T0	T1			T0	T1		
	M (SD)		*t*	*p*	M (SD)		*t*	*p*
**Primary**								
HDRS	15.75 (3.78)	11.50 (1.66)	3.40	<.05	15.75 (3.78)	11.50 (1.66)	2.75	.07
**Secondary**								
C-SHAPS	40.75 (3.82)	43.25 (2.78)	N.A		40.75 (3.82)	43.25 (2.78)	-3.00	.06
C-DARS	43.25 (7.23)	43.25 (8.26)	N.A		43.25 (7.23)	43.25 (8.26)	-.09	.94
SOFAS	58.00 (2.34)	59.25 (2.14)	N.A		58.00 (2.34)	59.25 (2.14)	-1.00	.39
GAF – Role	6.00 (0.00)	6.00 (0.00)	N.A		6.00 (0.00)	6.00 (0.00)	N.A	
GAF – Social	6.00 (0.82)	6.00 (0.82)	N.A		6.00 (0.82)	6.00 (0.82)	N.A	
RFS – Work	3.50 (0.58)	3.50 (0.58)	N.A		3.50 (0.58)	3.50 (0.58)	-1.00	.39
RFS – Independent living	3.50 (0.58)	3.50 (0.58)	N.A		3.50 (0.58)	3.50 (0.58)	N.A	
RFS – Immediate social	3.75 (0.96)	3.50 (0.58)	1.00	.39	3.75 (0.50)	3.75 (0.50)	N.A	
RFS – Extended social	3.75 (0.96)	3.75 (0.96)	N.A		3.25 (0.50)	3.25 (0.50)	N.A	

Note. Paired sample t-Tests were conducted. T0: Baseline; T1: Post-intervention. HDRS: Hamilton Depression Rating Scale; C-SHAPS: Chinese version of the Snaith-Hamilton Pleasure Scale; C-DARS: Chinese version of the Dimensional Anhedonia Rating Scale; SOFAS: Social and Occupational Functioning Assessment Scale (GAF)-Role/-Social: Role Functioning Scale (RFS)-work/-independent living/-immediate social/-extended social
